# New Appliances and Things Medical

**Published:** 1899-01-28

**Authors:** 


### NEW APPLIANCES AND THINGS MEDICAL.

[We shall be glad to receive, at our Office, 28 & 29, Southampton Street, Strand, London, W.C., from the manufacturers, specimens of all new preparations and appliances which may be brought out from time to time.]

### DAHL'S STERILISED DOUBLE CREAM.

(LONDON AGENTS: HERBERT FRANCIS AND CO., 8, ARTHUR STREET WEST, LONDON, E.C.)

THIS cream is carefully preserved in hermetically sealed tins after complete sterilisation. It may therefore be regarded as absolutely free from living germs of all kinds, including those of tuberculosis. This latter is a most important consideration, especially in the nursery and for invalids. The cream, which should be thoroughly whisked before using, is indistinguishable in consistency from ordinary fresh cream, and in flavour it loses little by the exposure to the temperature which is necessary for the sterilisation. For cooking it will be found as economical as for nursery purposes it is satisfactory.

### ELLIS'S PATENT SYPHON.

(R. ELLIS AND SON, RUTHIN.)

THIS new form of syphon, which has recently been patented by Messrs. Ellis, the well-known manufacturers of table waters, of Ruthin, in North Wales, has interesting features about it which make it well worthy of notice. The object of the new arrangement is to get rid of several well-recognised evils which seem inseparable from the syphon in its ordinary form. In the first place, the ordinary syphon is filled and emptied by way of the same orifice, and, should any dust or dirt have collected in the outlet spout, the act of filling it forces all these extraneous matters back into the syphon. Then, unless the entire head is removed, its mechanism is inaccessible for purposes of repair, as also is the vase itself for any effectual cleaning. In the new apparatus, however, the head forms a sort of cross, something like the top of a crutch walking-stick, and while the aerated water comes out at what we may call the long arm of the crutch, the fresh charge is inserted through the short arm, which, as soon as the charge is in, is firmly closed by means of a screw cap. Thus there is no to and fro current, the water always passes through in the same direction; no accumulation of dirt in the pipe can be carried back into the glass vessel, and for cleaning purposes it is possible to send a stream of water right through the whole apparatus. It is a handsome-looking syphon, it is easy to lift by the head, and the arrangement for the discharge of the aerated water is very satisfactory in its action. We are, however, principally attracted by its sanitary advantages. Knowing as we do where syphons go and what they are used for, a syphon which cannot have pumped back into it the contents of the spout is an immense improvement.

### PHENALGIN.

(ETNA CHEMICAL COMPANY, 313, WEST STREET, NEW YORK, U.S.A. LONDON AGENT: E. J. REID, 11, DUNEDIN HOUSE, BASINGHALL STREET, E.C.)

THIS new synthetic coal-tar preparation is reputed to possess very remarkable anæsthetic and analgesic properties. Its chemical constitution (ammorio phenylacetamide) shows that it belongs to that large group of antipyretics and analgesics which have become so popular during the last decade, and which are similarly the result of laboratory synthesis. Phenalgin, however, differs from them in one important respect at least, in that it is an ammoniated derivative. The combination of the ammonia radicle led its supporters to anticipate a certain degree of cardiac stimulation after its administration, and although precise physiological experiments do not appear as yet to have undertaken to prove this point, there has resulted abundant clinical evidence to prove that this is actually the case. This is a most important feature, since, as is well known, a certain amount of cardiac depression has occasionally followed on the administration of ordinary does of antifebrin, antipyrin, &c., and even in some cases alarming symptoms have supervened. Hence in cases of fatty heart or cardiac weakness their use is rightly contra-indicated. Phenalgin is a white crystalline body with a strong ammoniacal taste and odour. It may be used in combination with other drugs.

